# Self-Medication Practice in Limmu Genet, Jimma Zone, Southwest Ethiopia: Does Community Based Health Insurance Scheme Have an Influence?

**DOI:** 10.1155/2018/1749137

**Published:** 2018-02-20

**Authors:** Bayu Begashaw Bekele, Shibiru Tesema Berkesa, Enyew Tefera, Abera Kumalo

**Affiliations:** ^1^Department of Public Health, College of Health Sciences, Mizan Tepi University, Mizan Aman, Ethiopia; ^2^Institute of Public Health, College of Medicine and Health Sciences, University of Gondar, Gondar, Ethiopia; ^3^Department of Pharmacy, Jimma University, Jimma, Ethiopia; ^4^Medical Laboratory Sciences Department, College of Medicine and Health Sciences, Wolaita Sodo University, Sodo, Ethiopia

## Abstract

**Background:**

Self-medication, which is a form of self-care, is an important initial response to illness, and many illnesses can be successfully treated at this stage. It is practiced by a considerable proportion of the population and is affected by sociodemographic and economic factors. This study was conducted to assess the practice of self-medication and associated factors in Limmu Genet's town households, Jimma Zone, Southwest Ethiopia.

**Methods and Materials:**

A community based cross-sectional study was done. Systematic sampling technique was used to select participants. Data was collected by face-to-face interviews by using structured questionnaires. After checking the completeness, missing values, and coding of questionnaires, data was tabulated and calculated on SPSS version 20.0. Finally data was presented in tables, graphs frequency, percentage, and cross-tabulation with different variables.

**Result:**

In this study, both self-medication and the prevalence of diseases among households were 78.1%. That constituted any kind of illness reported by participants.

**Conclusion:**

Self-medication practice is common among community members regardless of being community based health insurance members. Therefore, it needs pertinent health education on legal prescriptions and use of medicines as well as strengthening the access of community based insurance.

## 1. Introduction

### 1.1. Background

Providing useful and effective healthcare to the community is essential to keep good health as well as a vital agenda of life. In low- and middle-income countries (LMICs), rural inhabitants especially have poor access to modern healthcare infrastructures and low affordability of drugs and medical supplies [1]. Therefore, most people acquire diseases easily. Consequently, episodes of illness are haphazardly treated by self-medication. But the means of self-treatment vary from person to person depending on several factors. The main care of illness is self-care, the oldest and most abundantly used behavior that affects the health of individuals either negatively or positively [1, 2].

Thus, self-medication can be defined as the use of drugs to manage self-diagnosed health complaints or symptoms or the usual use of a prescribed drug for chronic or acute diseases or disease manifestations. According to some studies, the demand of patients is increasing for efficient drugs available without prescription [3, 4]. However, inappropriate self-medication leads to resources wastage, increases microbial resistance, and could generally result in serious health hazards such as adverse drug reaction and persistent suffering [5].

Medicines for self-medication are often called “nonprescription” or “over-the-counter” (OTC) medicines, which are claimed as safe, effective for use, and available without a medical professional's prescription. In some countries, OTC products are available and dispensed by supermarkets and other outlets. On the other hand, medicines that require a doctor's prescription are called prescription products [6].

Self-medication is a form of self-care, which is basically performed to treat illnesses, with the ability to relieve illnesses at this stage. Self-medication is used by a significant number (proportion) of the population. It is affected by socioeconomic and demographic health facilities and economic factors. Although some healthcare providers exhibit negative understanding and perspectives towards self-medication, the World Health Organization (WHO) recognizes the existence of a valid role of it. There are a number of reasons why self-medication gets focus. The transition of the trend of diseases especially chronic ones with attendant shift from cure to care is becoming a large-scale transition. In addition, the inaccessibility (failures) of the healthcare system with its uneven distribution of drugs, unaffordable price, and the issue of curative stance of drugs is notable [7, 8]. Also, patient-related factors anticipate inappropriate antimicrobial use and contributed to the increasing prevalence of antimicrobial resistance (AMR). For instance, self-medication with antimicrobials by patients contributes to the problem of AMR [9]. Moreover, it might be associated with some risks such as drug interactions, adverse drug reactions, increased polypharmacy, incorrect diagnosis, and drug dependence [3, 10, 11].

Community based health insurance (CBHI) is defined as a mechanism that households in a community (the population in a village, a district, or other geographical areas, or a socioeconomic or ethnic population group) use to finance or cofinance the current and/or capital costs associated with a given set of health services and enable them to have some involvement in the management of the community financing scheme and organization of health services [12]. It aims to improve access to healthcare and to protect households from pocket-payment related risks. It incorporates a wide range of nonprofit schemes providing risk pooling to cover a part of or all healthcare related costs. To their minimum or maximum level, CBHI schemes target households that generate their income from the informal economy and are excluded from legal and formal social protection. Also, having a membership is therefore usually voluntary. Basically, the schemes encourage active decision-making and management. Consequently, building on mutual aid and unity, risk sharing is as inclusive as possible and membership premiums are independent of the individual health status. Nevertheless, payments are usually flat rates and, thus, are not adjusted and are requested according to the financial capacity of individuals [13, 14]. Therefore, being a CBHI scheme member might be one of the elements for self-medication prevalence in the study area.

Moreover, limited access to health information and education on medications and diseases might be linked with hazardous drawbacks to communities in rural areas. Lack of trained pharmacists/professionals creates favorable environment for self-medication. Even though SMA is somehow important to treat minor ailments, wrong self-medication practice may cause serious adverse drug reactions and possible fatal consequences. For instance, currently, AMR is becoming globally prevalent, mainly in LMICs [15]. For this reason, the transmission of new forms of resistant pathogens could be fast and can easily spread intercontinentally. World health leaders have described AMR as “nightmare bacteria” that “pose a catastrophic threat” to people in every country in the globe [16]. Therefore, the issues of drug availability, preference, and proper use are a vital issue to the global community [17].

However, illegal scandalmongers of drugs are prevalent in several LMICs. Thus, the use of these drugs from informal sources increases the chances of self-medication and home drug storage. The availability of drugs at home has enabled an extent of self-medication for illnesses. For that reason, handling unnecessary dangerous and bacterial resistant medicines from illegal and informal sources is important to consider the manners of drug availability to consumers [18].

As per studies in Ethiopia, the magnitude of self-medication varied from 12.8% to 77.1%, with an average of 36.8%. The type of illness that leads to self-medication, sources of information for self-medication, and drugs or category of drug products that are commonly self-administered need to be understood to design interventions [7, 19]. Some recent studies also revealed that antibiotics can be easily purchased without prescription and are accessible from* kiosks* (small local shops in Ethiopia). In addition, self-medication is still used more often for self-limiting illnesses and antibiotics are usually used by consumers. All these scientific findings have shown that antibiotics are not only highly consumed but also irrationally used. They also indicate that Ethiopia still faces some challenges related to regulatory enforcement from socioeconomic, demographic, and patient-related health facilities and policy related concerns [2, 7, 19, 20].

A study conducted in Butajira (Southern Ethiopia) indicated that 15% of people with perceived illnesses performed self-medication [21]. In another study conducted in Addis Ababa and Central Ethiopia, the magnitude of self-care was as high as 50%. Low severity/pain of illness or diseases and poverty were the major reasons for self-medication [2].

In previous studies, the association between being a CBHI member and the self-medication trend has not been studied yet in Ethiopia. Therefore, this study aimed to assess the prevalence and the nature of these practices in the CBHI members and nonmembers levels, which is important to devise appropriate educational, regulatory, and administrative measures.

## 2. Methods and Materials

### 2.1. Study Setting

A community based comparative cross-sectional study was conducted in a community in Limmu Genet town from February to March 2016, because in the town there are a number of private pharmacies and governmental hospitals which provide both preventive and curative services for communities in and out of the catchment areas. Limmu Genet is located 425 kms and 75 kms southwest of Addis Ababa, the capital of Ethiopia, and Jimma, the capital of Jimma Zone, respectively. It has a total surface area of 1200 hectares. It is bounded by Sokoru in the east, Gomma in the west, Limmu Sakka in the north, and Kersa in the south. The total projected population of the town from the 2007 Central Statistical Agency (CSA) census report is 12,674. The town has a temperature within the range of 30°C and an average annual rainfall of 800–2500 mm^3^ and is at an altitude of 1750–2000 m above sea level.

### 2.2. Participants, Sample Size, and Sampling Techniques

All households found in Limmu Genet town during the study period were used as the source population of the study. The study population was selected from community health insurance members and nonmembers, found in Limmu Genet town during the study period, who were regarded as the source population of the study. Based on eligibility criteria of households, members and nonmembers of CBHI were the study units. All community health insurance members and nonmembers were included in the study. All community health insurance members aged below 18 years were excluded. After this, 422 study participants were recruited in the study.

The multistage systematic sampling technique was used to select participants for data collection from the community. After the sample size was determined, the systematic random sampling technique was applied. Systematic random sampling was used to select study participants.

### 2.3. Data Collection Procedure and Instruments

Data collection tools were adapted from various literatures after a thorough revision of them. The questions and statements were grouped and arranged according to the particular objectives that they could address. The instruments included both closed-ended and open-ended questionnaires.

Data was collected on self-medication for both the health insurance members and the nonmembers. One questionnaire took an average of 20–30 minutes for a person. Patient characteristics such as sociodemographics and history of drug use were recorded.

### 2.4. Data Analysis

Data were analyzed using SPSS version 20. Descriptive statistics were used to illustrate proportions, tables, and means. Rates of total self-medication were calculated for each of the variables considered. Statistical analysis was carried out using Pearson's CBHI square with values of *P* < 0.05 taken as significant.

To estimate the independent effect of each of these variables on self-medication, further statistical analysis was carried out by linear regression. Initially, data showed that being a member of community insurance was found to be an important factor in self-medication and, in turn, community insurance might have influenced other variables.

### 2.5. Quality Control

There was regular cross-checking for the completeness of the questionnaires. The data collection tool was pretested for its validity. The data collection tool was first prepared in English and later translated to Afan Oromo local language to keep meaning consistency. To avoid information bias, pretest was conducted in the adjacent district town, and after getting feedback from the pretest we revised the tool to maintain validity.

### 2.6. Ethical Clearance

The ethical approval and clearance letter of permission was obtained from Jimma University; an official letter was obtained from the Department of Pharmacy. During data collection, all respondents were asked for their permission and informed consent was obtained prior to the interview. An official letter was sent to Limmu Genet's health center and hospital. The confidentiality of the study participants was secured.

## 3. Results

### 3.1. Sociodemographic Characteristics of the Respondents

This population-based survey examined three hundred and eighty-nine households which were sampled from three kebeles. Of the total 422 questionnaires distributed to be filled by respondents, 389 were filled completely and collected, which gave a response rate of 92.2%. The mean age of the respondents was 42.2 years (SD = 13.7). The minimum age was 18 and the maximum was 90. Since the interview was conducted with female household heads, 47.6% of the respondents were females. Regarding the respondents' family monthly income, the majority, 37.0%, reported a monthly income of between 1001 and 1500 Ethiopian Birr (Table 1).

### 3.2. Characteristics of the Sick Person

From a total of 389 households, three hundred and eight (308) household members had faced health-related problems within the last two weeks prior to the study, translating to an illness prevalence of 91.8%. But the degree of illness varies from person to person. Among them, 241 (78.2%) treated themselves. No households reported more than one ill person. About 95.4% of the sick individuals were married. Females, 48%, reported less illness than males, 52%.

### 3.3. Perceived Illness Treated with Self-Medication with Drugs

Depending on the different socioeconomic and sociodemographic factors, the types, extents, and reasons for self-medication can vary from country to country. In this study, self-medicated participants were 237 with health problems out of 304 with illness conditions. This made self-medication's prevalence 78.1%. Over 70% of CBHI members and 30% of nonmembers practiced self-medication. The most common types of ailments for which the respondents reported practicing self-medication were cough and fever (Table 3).

### 3.4. Extent and Reason for Self-Medication with Drugs

The major reasons listed by the self-medicated study participants are shown in Table 2. Concerning reasons of self-medication respondents, about 114 (67%) and 63 (70%) of CBHI members and nonmembers, had low cost alternatives in money and time, respectively. But only 6 (3%) of CBHI members had prior experience to the illness and/or the self-drug intake. About 56 (26%) and 21 (23%) CBHI members and nonmembers believed that self-medication is an emergency care, respectively.

### 3.5. Major Drug Classes Used for Self-Medication

**Figure 1:**
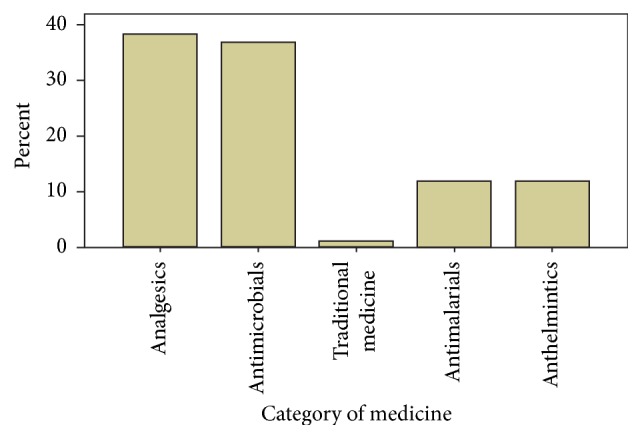
Categories of drugs requested for self-medication (*n* = 241) in Limmu Genet community, May 2016.

Out of 241 self-medicated participants, the most commonly requested categories of drugs were analgesics/antipyretics (93, 38.5%), antimicrobials (91, 36.8%), antimalarials (28, 11.8%), anthelmintics (28, 11.8%), and traditional medicine (3, 1.0%) (Figure 1).

### 3.6. Sources of Information for the Practice of Self-Medication with Drugs

The two most usual sources of advice/information for self-medication were drug retail outlets (37.8%) and healthcare providers such as doctors, nurses, and health assistants, but without formal prescriptions (42.1%). Nevertheless, friends, neighbors, or relatives (2%) and 37.8% of the respondents obtained information by reading drug-related materials such as labels, leaflets, or promotional materials, while 18.0% of them reported obtaining such information by previous experience (Table 4).

### 3.7. Determinants of Self-Medication with Drugs

As shown in Tables 5 and 6, the result of crosstabs analysis revealed that, in some variables, significant associations were observed on the practice of SMA and community health insurance members and nonmembers.

## 4. Discussion

Self-medication in our study was much higher than it was in previous studies. Mainly, the increasing healthcare cost and the shift in the pattern of diseases towards chronic ones (from 30% to 80% in 40 years) led to a more person-centric approach involving self-care and responsible self-medication [6, 22]. Recent reports from developed countries clearly show extensive use of self-medication. Worldwide studies also showed a range of self‐medication practices between 15% and 80% [1, 23].

In this study, the prevalence of self-medication was found to be 78.2%, which is almost more or less similar to other studies in the country [1, 23]. However, this result was higher than the results of some other studies done in Ethiopia [7, 18, 20, 24]. It is more than three times higher than the findings from Spain and India. But categorically, 79.6% of the CBHI members and 75% of the nonmembers practiced self-medication. Even though the practice of self-medication varies across countries, it is difficult to make any comparative analysis since there is no prior study conducted in an area similar to the one under study. Therefore, the higher proportion differences observed among different groups might be due to free access of drugs for members and charged drugs for nonmembers. In addition, nowadays, people may be aware of some drugs indications for usual and prevalent illnesses like analgesics for headache and antimalarials for malaria. Consequently, the prevalence of self-medication might be high if people are aware of those drug uses.

This study also showed that 37% of the study population used antibiotics without medical prescription. Recent reports from developed countries clearly show that SMA is commonly encountered. Our findings are in line with the study from Israel in which 37% of participants treated themselves with antimicrobial drugs. However, this proportion was too much higher than the ones in Northern Europe (3%), Central Europe (6%), and Southern Europe (19%). This was lower than findings from Pakistan (69%), China (48%) [26], Sudan (one-month period prevalence was 74%) [27], Yemen (60%) (within two-week recall period) [28], and Ghana (70%) [1]. This might be due to the socioeconomic difference between settings. Still we were not able to compare between two groups of CBHI due to unavailability of a similar study elsewhere. This might be increased with bacterial diseases prevalence such as diarrheal and abdominal discomfort, which might lead inhabitants to use antibiotics on their own without any prescription and regardless of being a CBHI member.

Higher period prevalence has been reported in most of the developing countries. This has been associated with several factors, particularly the lack of access to healthcare, availability of antibiotics as over-the-counter drugs, and the relatively higher prevalence of infectious diseases [1]. In this study, the two-week period prevalence of SMA is higher than in the previous recent studies done in Ethiopia (a three-month period prevalence of 6.7% [18] and a two-month period prevalence of 4.8% [29]). This variation could be attributed to the recall period used in the studies or variation in the study areas.

Even if direct studies on the availability of antibiotics without prescription in pharmacies in Ethiopia are scarce, several studies have shown that the main source of drugs that are used for self-medication was pharmacies [18, 20, 24, 29].

The present study result also showed that 42% of the individuals who practiced SMA reported that they obtained drug-related information from other health professionals. Also, they obtained drug-related information (at least when to take and what should never be taken with the drug) from the dispensers. This is not in agreement with studies done in most developed countries where leftovers from previous courses and from relatives were the main sources of drugs for the practice of SMA [25, 26]. This implies that healthcare professionals take care of information regarding the right place, route, time, dose, and frequency of medication while dealing with it.

While it was consistent with what has been reported in previous studies in Africa [1, 15, 27, 28], a similar study in India also found that 20% of the antibiotics are purchased without a doctor's prescription [30].

Findings from key informants with private community pharmacists also showed that private pharmacies were the major source of drugs for self-medication. All of them reported that people could buy antibiotics without a prescription, and when antibiotics are requested by consumers, requests are neither refused nor questioned. Though antibiotics are prescription-only drugs, community pharmacists dispense these drugs without a doctor's prescription. This might lead to the misuse or overuse of antibiotics. This irrational use of antibiotics promotes antibiotic resistance [31]. The consequence of this is the switch from relatively cheap drugs to new drugs, which will be more expensive for developing countries such as Ethiopia. Overall, this indicates that there is a weak regulatory system in the study area. In addition, pharmacists should respect their professions.

Previous experience with similar symptoms and mildness of the illness were the two major reasons for SMA in this study. The main reasons for SMA in developing countries include OTC sale of antibiotics, high cost of medical consultation, low satisfaction with medical practitioners, and misconceptions regarding the efficacy of antibiotics [30]. The implication of prompt consideration is important regarding affordability and availability of drugs and medical supplies in public health facilities.

The present study results showed that some sociodemographic variables had a significant association with the practice of SMA. This finding is consistent with several study results in Africa and Ethiopia where the educational level is a significant factor for the practice of SMA [18, 21, 30]. Similar researches conducted in Sudan and Yemen also showed that the risk of SM was higher among females compared to males or that females had a higher risk of self-medication behavior compared to males [27, 28].

The prominent limitations of this study were the lack of previous comparative, cohort, or RCT studies, which made the discussion poor. Also, the recall bias might affect the prevalence and type of drug used by the participants. In addition, participants were asked simply for self-medication but not for the type of drugs they used. This might make sense for all types of drugs used by them.

## 5. Conclusion

There is no significant difference between members and nonmembers of CBHI concerning self-medication in the study area. Although appropriate self-medication can be advantageous without proper education of the public and proper regulation of potent drugs dispensary, it may cause tragic consequences. Thus, health education regarding the dangers of self-medication must also be given with impressive attention.

Therefore, education on the prescription and use of medicines as well as strengthening the CBI scheme utilization among the society is needed. Further interventional studies are needed to evaluate the effect size of being a CBHI member versus self-medication in the study area.

## Figures and Tables

**Table 1:** Sociodemographic characteristics of respondents (head of the household), Limmu Genet town, May 2016 (*N* = 389).

Sociodemographic profile	*N* (%)
CBI	
Members	269 (69%)
Nonmembers	120 (31%)
Sex	
Male	204 (52%)
Female	185 (48%)
Age	
<18	9 (2%)
19–35	133 (34%)
36–59	187 (48%)
>60	60 (16%)
Marital status	
Married	357 (92%)
Single	18 (5%)
Widowed	5 (1%)
Divorced	9 (2%)
Religion	
Orthodox	121 (31%)
Muslim	265 (68)
Protestant	3 (1%)
Educational status	
Illiterate	139 (36%)
Grade 1–8	197 (51%)
Grade 9–12	43 (11%)
Higher education	10 (3%)
Ethnicity	
Oromo	310 (80%)
Amhara	54 (14)
Tigre	7 (2%)
Other	18 (5%)
Monthly income	
<550	47 (12%)
501–1000	63 (16%)
1001–1500	144 (37.0%)
1501–2000	120 (31%)
>2000	15 (4%)

**Table 2:** Sociodemographic characteristics of those who reported an illness within the 2-week recall period, Limmu Genet town, May 2016 (*N* = 241).

Sociodemographic profile	*N* (%)
CBI	
Members	308 (77%)
Nonmembers	90 (23%)
Sex	
Male	157 (52%)
Female	147 (48%)
Age	
<18	5 (2%)
19–35	98 (32%)
36–59	158 (52%)
>60	43 (14%)
Marital status	
Married	290 (96%)
Single	7 (2%)
Widowed	4 (1%)
Divorced	3 (1%)
Educational status	
Illiterate	115 (38%)
Grade 1–8	197 (54%)
Grade 9–12	21 (5%)
Higher education	4 (1%)
Monthly income	
<550	23 (78%)
501–1000	30 (10%)
1001–1500	129 (42%)
1501–2000	114 (38%)
>2000	8 (3%)

**Table 3:** Type of illness reported (*N* = 241) and action taken by the study participants, May 2016, Limmu Genet town.

Type of illness	Community based health insurance members, *N* (%)	Community based health insurance nonmembers, *N* (%)	Total, *N* (%)
Headache	25 (68.9%)	11 (31.1%)	36 (14.8%)
Fever	35 (66.7%)	17 (33.3)	52 (21.7%)
Cough	38 (70.6%)	16 (29.4)	54 (22.4%)
Diarrhea	37 (73.0%)	14 (27.0)	51 (20.7%)
Others	34 (72.9%)	14 (27.1%)	48 (19.4%)

*Total *	*170 (70.4%)*	*71 (29.6%)*	*241 (100%)*

**Table 4:** Reasons for self-medication in community in Limmu Genet town (*N* = 241), May 2016.

Community based health insurance	Reason for self-medication				Total
	Low cost alternative in money and time	Illness was minor (not serious)	Emergency	Previous experience of drug use	
Member	114 (67.3%)	6 (3.7%)	45 (26.2%)	5 (2.8%)	170 (70.4%)
Nonmember	50 (70%)	5 (6.7%)	16 (23.3%)	0 (0%)	71 (29.6%)

*Total*	164 (68.0%)	11 (4.6%)	61 (25.4%)	5 (2.0%)	241 (100%)

**Table 5:** Sources of information or advice for self-medication at Limmu Genet community (*n* = 241), May 2016.

Community based health insurance	Source of information				Total
	Drug retail outlet	Other health professionals	Previous experience	Neighbor	
Member					
Number	68	62	36	4	170
Nonmember					
Number	23	39	8	1	71

*Total *	91	101	44	5	241

**Table 6:** Association made between CBHI members and nonmembers to self-medication reported by selected background variables, May 2016 in Limmu Genet (*n* = 241).

Variables	Self-medication	CBHI square	*P* value
Marital status			
Married	230 (95.4%)	*X* ^2^ = 28.8	*P* > 0.05
Single	6 (2.3%)		
Widowed	4 (1.6%)		
Divorced	1 (0.98%)		
Educational status			
Illiterate	91 (37.8%)	*X* ^2^ = 34.8	*P* < 0.000
Grade 1–8	130 (53.9%)		
Grade 9–12	17 (6.9%)		
Higher education	4 (1.3%)		
Monthly income			
<550	18 (7.5%)	*X* ^2^ = 9.08	*P* > 0.059
501–1000	24 (9.8%)		
1001–1500	102 (42.4%)		
1501–2000	90 (37.5%)		
>2000	6 (2.6%)		
Occupation			
Farmer	121 (50%)	21.98	*P* < 0.000
Merchant	81 (33.8%)		
Government employed	9 (2.9%)		
Daily laborers	23 (9.5%)		
Other	9 (3.6%)		

## Data Availability

All relevant data are within the paper and supporting information files but any additional data required by the journal can be available anytime.

## Abbreviations

AMR: Antimicrobial resistance
CBHI: Community based health insurance
GC: Gregorian calendar
JU: Jimma University
LMICs: Low- and middle-income countries
RCT: Randomized controlled trial
SMA: Self-medication with an antimicrobial
WHO: World Health Organization.
